# APOBEC3 mutational signatures are associated with extensive and diverse genomic instability across multiple tumour types

**DOI:** 10.1186/s12915-022-01316-0

**Published:** 2022-05-21

**Authors:** G. Maria Jakobsdottir, Daniel S Brewer, Colin Cooper, Catherine Green, David C Wedge

**Affiliations:** 1 Wellcome Centre for Human Genetics, University of Oxford, Roosevelt Drive, Oxford, OX3 7BN UK; 2 Big Data Institute, University of Oxford, Old Road Campus, Oxford, OX3 7LF UK; 3 Manchester Cancer Research Centre, University of Manchester, Wilmslow Road, Manchester, M20 4GJ UK; 4 University of East Anglia, Norwich Research Park, Norwich, NR4 7TJ UK; 5 Oxford NIHR Biomedical Research Centre, Oxford, OX4 2PG UK

**Keywords:** Mutational signatures, APOBEC3, Genomic instability

## Abstract

**Background:**

The APOBEC3 (apolipoprotein B mRNA editing enzyme catalytic polypeptide 3) family of cytidine deaminases is responsible for two mutational signatures (SBS2 and SBS13) found in cancer genomes. APOBEC3 enzymes are activated in response to viral infection, and have been associated with increased mutation burden and TP53 mutation. In addition to this, it has been suggested that APOBEC3 activity may be responsible for mutations that do not fall into the classical APOBEC3 signatures (SBS2 and SBS13), through generation of double strand breaks.Previous work has mainly focused on the effects of APOBEC3 within individual tumour types using exome sequencing data. Here, we use whole genome sequencing data from 2451 primary tumours from 39 different tumour types in the Pan-Cancer Analysis of Whole Genomes (PCAWG) data set to investigate the relationship between APOBEC3 and genomic instability (GI).

**Results and conclusions:**

We found that the number of classical APOBEC3 signature mutations correlates with increased mutation burden across different tumour types. In addition, the number of APOBEC3 mutations is a significant predictor for six different measures of GI. Two GI measures (INDELs attributed to INDEL signatures ID6 and ID8) strongly suggest the occurrence and error prone repair of double strand breaks, and the relationship between APOBEC3 mutations and GI remains when SNVs attributed to kataegis are excluded.We provide evidence that supports a model of cancer genome evolution in which APOBEC3 acts as a causative factor in the development of diverse and widespread genomic instability through the generation of double strand breaks. This has important implications for treatment approaches for cancers that carry APOBEC3 mutations, and challenges the view that APOBECs only act opportunistically at sites of single stranded DNA.

**Supplementary Information:**

The online version contains supplementary material available at (10.1186/s12915-022-01316-0).

## Background

The APOBEC3 (apolipoprotein B mRNA editing enzyme catalytic polypeptide 3) enzymes make up a family of closely related cytidine deaminases that target single stranded DNA, and characteristically result in the generation of mainly C >T mutations, with slight differences in their preferred sequence contexts [1]. APOBEC3 activity is thought to be responsible for two well defined single base pair substitution (SBS) mutational signatures termed SBS2 and SBS13 [2]. SBS2 is defined by C >T mutations at the TCX sequence context and is also associated with C >G mutations in the same context. SBS13 is primarily associated with C >G mutations at the TCT and TCA context, and to a lesser extent with C >T mutations. APOBEC3A/B/C/D/F/H act preferentially at a TCX context, whereas APOBEC3G acts mainly at a CCX con- text [1, 3]. The main role of the APOBEC3 enzymes is to restrict viral infections and the activity of retrotransposons [4]. The APOBEC3 enzymes, which were originally identified through their role in restricting HIV infection, increase the mutational burden in the virus, resulting in a loss of infectivity [1, 5]. APOBEC3s have also been found to target human T-lymphotropic virus-1 (HTLV-1), human endogenous retroviruses (HERV), Epstein-Barr virus (EBV), torque teno virus (TTV), parvoviruses, Kaposi sarcoma virus, vaccinia virus, simian foamy virus (SFV), murine leukaemia virus (MLV), herpes simplex virus-1 (HSV-1), and hepatitis B virus (HBV) [1, 3, 6].

Although the APOBEC3 enzymes have well defined roles in the cell, they have come under investigation as potential sources of cancer initiation and progression due to their off-target effects on the host genome. Overexpression of APOBEC3A in cellular systems causes DNA breaks, DNA damage responses, and cell-cycle arrest, and APOBEC3B causes base substitutions in the host genome [7, 8]. The carcinogenic potential of APOBEC3s has been highlighted in many different cancers including multiple myeloma, breast cancer, lung cancer, and urothelial carcinoma [9–16].

High levels of APOBEC3 mutations have been linked with poor prognosis in multiple myeloma, while being associated with better survival in urothelial carcinoma [10, 14]. High APOBEC3 expression levels have also been associated with better overall survival in cisplatin-treated urothelial carcinoma [13]. mRNA expression levels of APOBEC3A and APOBEC3B have been found to correlate with mutation burden and increased numbers of APOBEC3 mutations [9, 14].

Activity of the APOBEC3 enzymes has also been linked to various forms of genomic instability, such as kataegis, which is thought to be caused by the action of APOBEC3 enzymes at single stranded DNA exposed during resection of DNA at DNA strand breaks [12, 17]. The presence of APOBEC3 mutational signatures has been associated with specific translocations found in multiple myeloma [10]. However, a study on breast cancer genomes did not find any correlation between the number of copy number aberration (CNA) segments and enrichment of an APOBEC3 mutational signature [9, 10].

It has been suggested that APOBEC3 enzymes may play a more causative role in the generation of genomic instability by causing the formation of double strand breaks, either through the excision of uracils and cleavage of the abasic site on opposing strands, or through stalling of replication forks at single strand breaks [16]. The role of AID (activation-induced deaminase), which is closely related to the APOBEC3 family, in somatic antibody diversification, and its association with translocations in B cell tumours, lends credence to this model of APOBEC3 induced double strand breaks [18].

Previous work has largely focused on APOBEC3 activity in breast cancer, and has often been limited to exome sequencing data. In this study, we provide evidence that APOBEC3 causes an increased mutation burden and genomic instability via generation of double strand breaks, through analysis of whole genome sequencing data from 2451 samples across 39 tumour types in the Pan-Cancer Analysis of Whole Genomes Project (PCAWG) [19].

## Results

### Number of APOBEC3 mutations correlates with total mutation burden

**Fig. 1.**
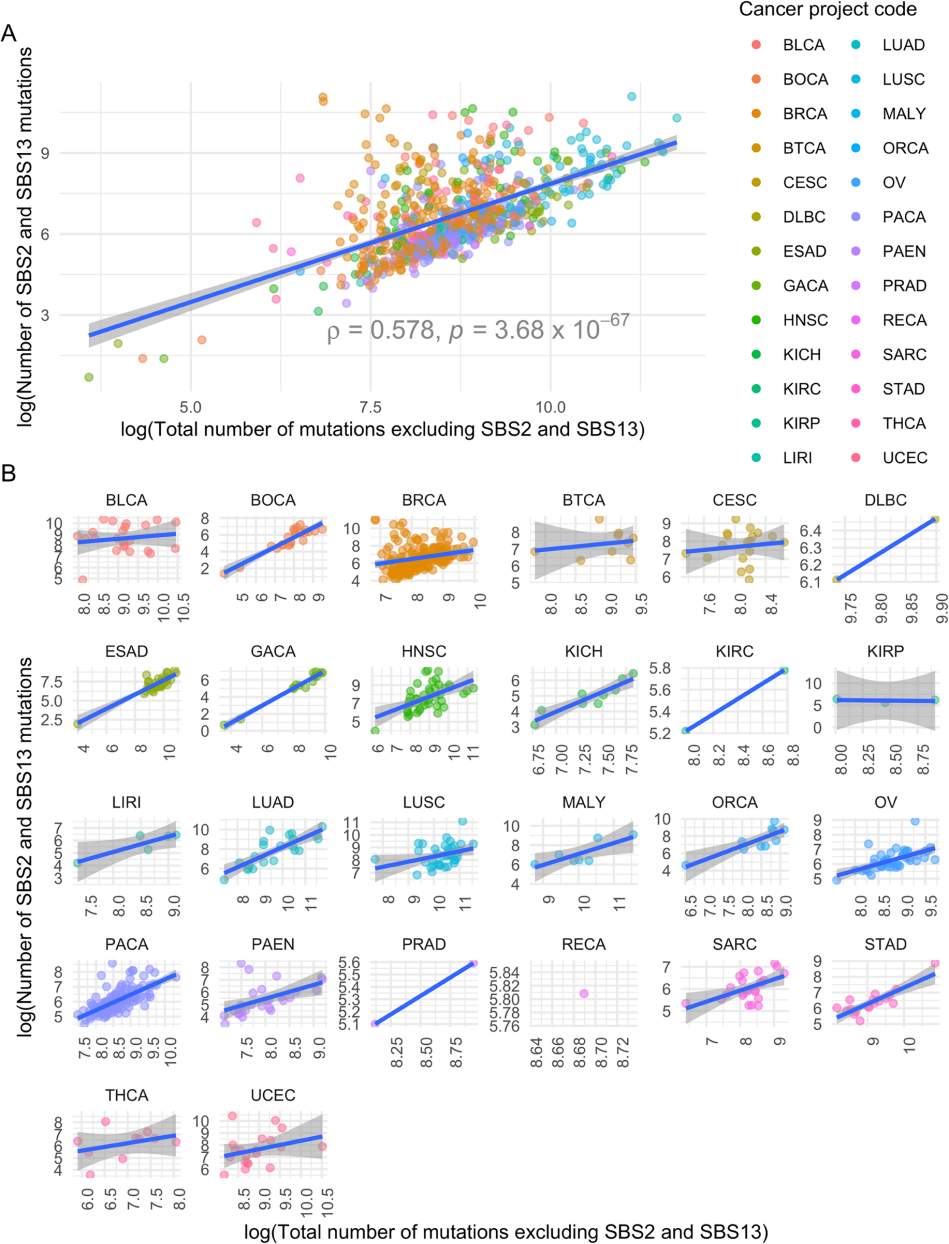
Correlation between number of SBS2 and SBS13 mutations and non-SBS2 and SBS13 mutations. **A** All tumour types. **B** Tumour types represented individually. Spearman correlation between the number of SBS2 and SBS13 SNVs and the total number of non-SBS2 and SBS13 SNVs for samples containing at least one SNV attributed to SBS2 and SBS13, coloured by tumour type and project code. The number of mutations was log transformed, using the natural logarithm. Shaded area represents the 95% confidence interval. Spearman’s *ρ* and *p* values for each of the correlations between the number of SBS2 and SBS13 and non-SBS2 and SBS13 SNVs by project code are presented in Additional file 1: Supplementary Table 1 (*n*=741)

We investigated the relationship between the number of classical APOBEC3 mutations (SBS2 and SBS13) and total mutation burden, excluding mutations attributed to SBS2 and SBS13. Of the 2451 primary tumours that we investigated, 741 (30.2%) were found to harbour mutations attributed to the APOBEC3 mutation signatures. Tumours carrying APOBEC3 mutations were found across 26 of the 39 tumour types included in the PCAWG data set (Fig. 1 and Additional file 1: Supplementary Table 1), and had a significantly higher mutation burden than tumours that did not carry APOBEC3 mutations (one-sided Wilcoxon rank-sum test *p* = 1.49 × 10^−26^). Further, the number of APOBEC3 mutations was significantly correlated with the total mutation burden for 14 of the 22 tumour types (63.6%), for which there were at least three samples available to calculate Spearman correlation from (Fig. 1 and Additional file 1: Supplementary Table 2), as previously observed in oral squamous cell carcinomas [20].

After taking into account the effect of tumour type, both age and the number of classical APOBEC3 mutations were significant predictors of the number of non-APOBEC3 SNVs (Mixed Effects model, *p* = 2.26 × 10^−3^ and *p* = 2.27 × 10^−49^, respectively. Additional file 1: Supplementary Table 3; Additional file 1: Supplementary Note 1) [21].

### Presence of APOBEC3 mutations is associated with increased genomic instability

It has previously been suggested that the increase in overall mutation burden coinciding with increased numbers of APOBEC3 mutations may arise through further processing of deaminated cytosines by DNA repair enzymes, resulting in the generation of transitions, transversions, and double strand breaks (DSBs) [16]. Errors in the repair of DSBs then result in mutations, as well as causing chromosomal rearrangements [16, 22]. Taking the number of APOBEC3 mutations as an indicator of previous APOBEC3 activity, we investigated their effect on multiple measures of genomic instability.

**Fig. 2.**
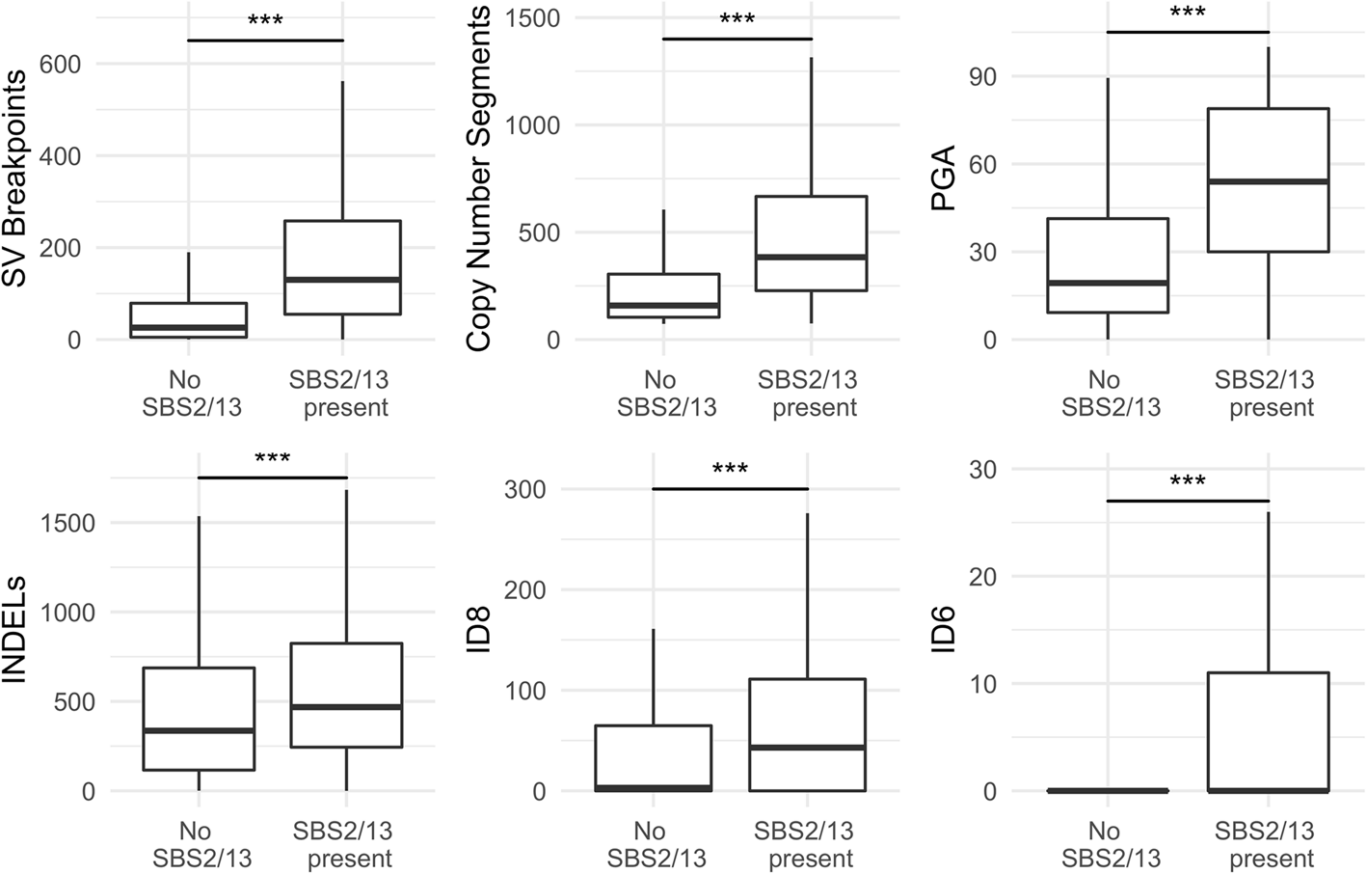
The effect of SBS2 and SBS13 presence on genomic instability. Measures of genomic instability by presence of SBS2- and SBS13-related signatures. PGA, Percentage of the Genome Altered. INDELs, Insertions and Deletions. ID8, insertion and deletion signature 8. ID6, insertion and deletion signature 6. (Wilcoxon Rank Sum test; * = *p* <0.05, ** = *p* <0.01, *** = *p* <0.001, *n* = 2451 for INDELs, ID8, ID6, PGA and Copy Number Segments. *n* = 2427 for SVs.). Individual *p* values are provided in Additional file 1: Supplementary Table 4

We used the number of structural variants (SVs), copy number (CN) segments, the percentage of the genome altered by copy number aberrations (PGA), and the number of insertions and deletions as measures of genomic instability. We also examined the number of insertions and deletions (INDELs) attributed to INDEL signatures 6 and 8 (ID6 and ID8), which have been associated with non-homologous end-joining (NHEJ) of double strand breaks (DSBs) [23]. For all six of the genome instability measures that we considered, samples carrying APOBEC3 mutations had significantly higher values than samples with no APOBEC3 mutations (Wilcoxon rank-sum test *p* <0.001; Fig. 2).

### The number of APOBEC3 mutations predicts the level of genomic instability across multiple tumour types

We constructed mixed effects models to investigate whether the number of APOBEC3 mutations could be used to predict the levels of the instability measures, taking both age and tumour type into account. Our models show that tumours carrying APOBEC3 mutations are more genomically unstable and that the number of APOBEC3 mutations is associated with all measures of genomic instability, except the number of ID6 INDELs (Table 1). Age had a significant predictive effect for the total number of INDELs and the number of structural variants (*p* = 9.61 × 10^−6^ and *p* = 0.0151, respectively).

**Table 1:** Mixed effects models predicting the levels of six different measures of instability using age, the number of SBS2 and SBS13 mutations, accounting for the effects of tumour type as a random variable. The number of SBS2 and SBS13 mutations was log transformed using the natural logarithm. These models correspond to models 3-9, detailed in Additional file 1: Supplementary Note 1. *SV* structural variant, *CN* copy number, *PGA* Proportion of the Genome Altered, *INDELs* Insertions and Deletions, *ID8* INDEL signature 8, *ID6* INDEL signature 6, *LMM* linear mixed effects model, *NB* negative binomial, *ZINB* zero inflated negative binomial, *AIC* Akaike Information Criterion)

	*Dependent variable:*						
		PGA	CN Segments	SVs	INDELs	ID8	ID6
	Model Type	LMM	NB	NB	NB	ZINB	ZINB
Count model: (Intercept)	Coefficient	3.62	4.42	0.397	1.36	0.514	5.46
	95% CI	(−9.49,16.7)	(4.01,4.83)	(−1.26,2.05)	(0.257,2.46)	(−1.52,2.55)	(1.30,9.63)
	*p* value	0.589	3.62*x*10^−99∗∗∗^	0.638	0.0157^∗^	0.620	0.0102^∗^
Count model: Age	Coefficient	0.0634	−0.000362	0.0323	0.0393	0.0250	−0.0562
	95% CI	(−0.0731,0.200)	(−0.00370,0.00298)	(0.00624,0.0583)	(0.0219,0.0567)	(−0.00703,0.0570)	(−0.121,0.0083)
	*p* value	0.363	0.832	0.0151^∗^	9.61*x*10^−6∗∗∗^	0.126	0.0877
Count model:	Coefficient	6.50	0.247	0.684	0.725	0.589	0.0191
log (SBS2/13)	95% CI	(4.99,8.01)	(0.206,0.289)	(0.442,0.927)	(0.562,0.888)	(0.281,0.897)	(−0.578,0.616)
	*p* value	2.51×10^−16∗∗∗^	5.66×10^−32∗∗∗^	3.14×10^−8∗∗∗^	3.43×10^−18∗∗∗^	1.77×10^−4∗∗∗^	0.950
Count model:	Coefficient			−0.00515	−0.00552	−0.00372	0.00738
Age:log (SBS2/13)	95% CI			(-0.00904,-0.00126)	(−0.00812,−0.00292)	(−0.00854,0.00110)	(−0.0019,0.0166)
	*p* value			0.00942^∗∗^	3.14×10^−5∗∗∗^	0.130	0.118
Zero model: (Intercept)	Coefficient					−0.684	1.07
	95% CI					(−0.839,−0.530)	(0.903,1.24)
	*p* value					4.20×10^−18∗∗∗^	3.85×10^−36∗∗∗^
AIC		6738.4	10,064.1	8701.2	10,444.3	6357.3	3270.9
Log likelihood		−3364.2	−5027.1	−4344.6	−5216.1	−3171.6	−1628.5
Num. obs.		725	725	717	725	725	725
Num. tumour type:		26	26	26	26	26	26

^∗^*p*<0.05; ^∗∗^*p*<0.01; ^∗∗∗^*p*<0.001

**Fig. 3.**
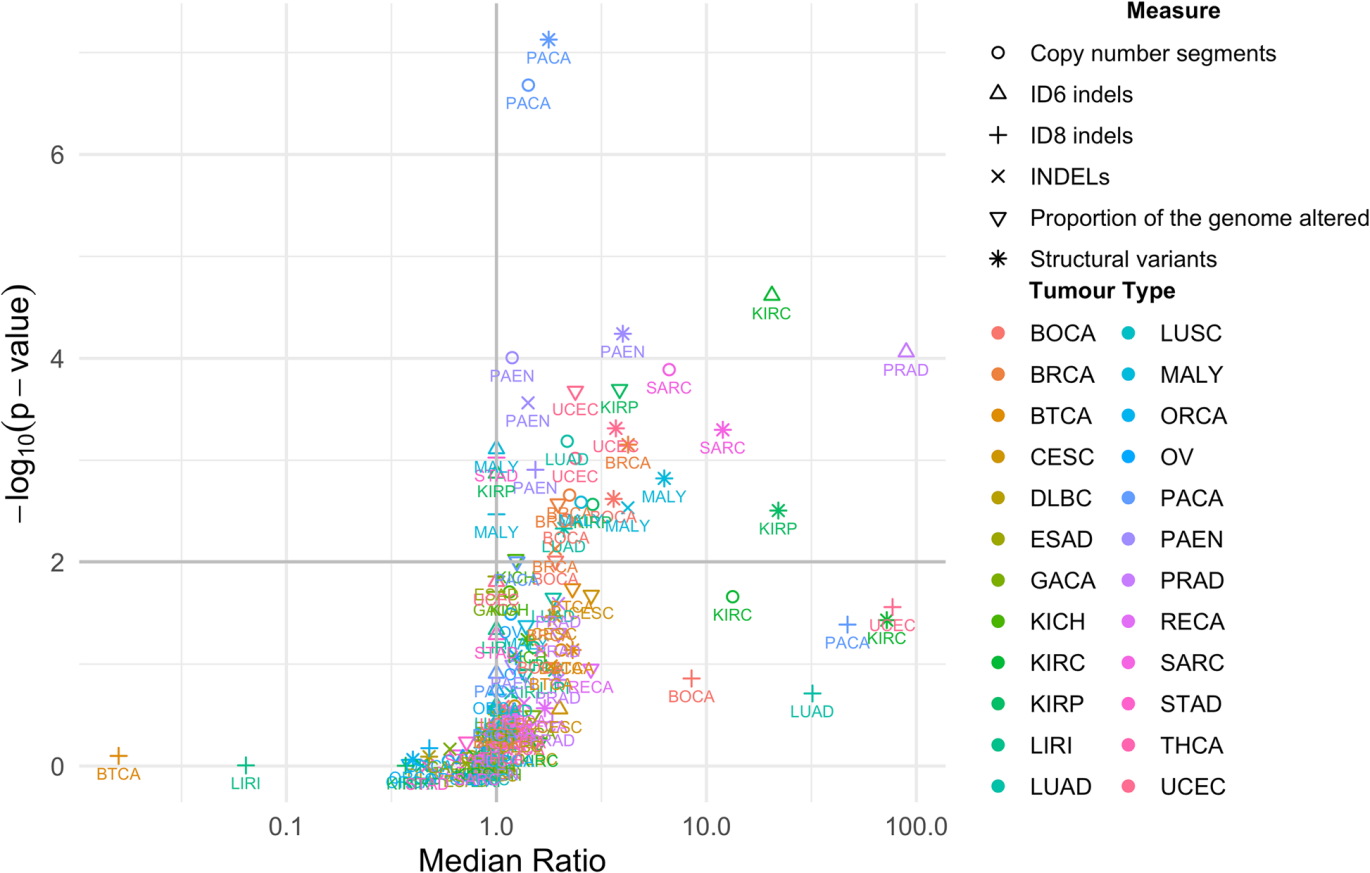
The effect of SBS2 and SBS13 presence on genomic instability by tumour type. Ratio of the median value of each measure of genomic instability for tumours containing SBS2 and SBS13 mutation to those that do not contain SBS2 and SBS13 mutations. *p* values were derived from one-sided Wilcoxon rank sum tests, and the horizontal grey lines indicates an FDR of 0.05, which includes points that fall on the line. The number of samples in which SBS2 and SBS13 mutations are present and absent are reported for each tumour type in Additional file 1: Supplementary Table 1. Details of the means and median ratios, and *p* values for each of the tumour type and genomic instability measure combinations are presented in Additional file 2: Supplementary Data 1

Comparing the median values for each of the six measures within a given tumour type highlighted several tumour types in which the presence of APOBEC3 mutations had a strong effect on genomic instability (Fig. 3). When individual measures of genomic instability are considered, 13 of the 24 tumour types (54.2%) had significant association between presence of APOBEC3 mutations and a measure of genomic instability. Specifically, higher levels of genomic instability were observed across multiple measures in tumours that contained APOBEC3 mutations than those that did not for both pancreatic cancer subtypes (Pancreatic Cancer Endocrine Neoplasms (PAEN) and Pancreatic Cancer (PACA)), Bone Cancer (BOCA), Kidney Renal Papillary Cell Carcinoma (KIRP), and Malignant Lymphoma (MALY). In addition, significant associations were observed for a single measure of genomic instability for Breast Cancer (BRCA), Lung Adenocarcinoma (LUAD), Kidney Renal Clear Cell Carcinoma (KIRC), Kidney Chromophobe (KICH), Gastric Adenocarcinoma (STAD), Uterine Corpus Endometrial Carcinoma (UCEC), Sarcoma (SARC), and Prostate Adenocarcinoma (PRAD). When we combined *p* values for all measures of genomic instability, a further 2 tumour types, Biliary Tract Cancer (BTCA) and Cervical Squamous Cell Carcinoma (CESC), showed significant association between presence of APOBEC3 mutations and GI (62.5%, Fisher’s combined probability test with Benjamini-Hochberg correction for multiple testing *p* value <0.05; Additional file 1: Supplementary Note 2; Additional file 1: Supplementary Table 5) [24].

### Both presence of APOBEC3 mutations and TP53 mutation affect genome stability

Several studies have found that activity of APOBEC3 proteins is intimately linked with p53 activity, with p53 acting as a negative regulator of APOBEC3B activity [25, 26]. In addition, APOBEC3 activity has been associated with mutations in the TP53 gene [16]. To further investigate this link, we built new models adding the effects of TP53 alterations.

The proportion of tumours carrying missense or nonsense mutations in TP53 was significantly higher in tumours carrying APOBEC3 mutations (41.6%) than in tumours not carrying any APOBEC3 mutations (19.9%; one-sided Fisher exact test, *p* = 9.91 × 10^−28^). Tumours carrying missense or nonsense mutations in TP53 also had a higher number of APOBEC3 mutations, as well as a higher non-APOBEC3 mutation burden (one-sided Wilcoxon rank-sum test, *p* = 3.53 × 10^−67^).

Adding the TP53 mutation status of the tumours to the mixed effects models generated in the previous section suggests that TP53 mutation is a significant predictor of the genomic instability measures, with the exception of the number of ID8 INDELs (Table 2). Importantly, the number of APOBEC3 mutations remained a highly significant predictor throughout, and also emerged as a significant predictor for the number of ID6 INDELs. For PGA, the number of copy number segments, the number of structural variants, and the number of ID6 INDELs, including TP53 in the model improved it significantly, but not for the total number of INDELs or ID8 INDELs (ANOVA *p* <0.05, Additional file 1: Supplementary Table 6). The effects of age on the measures of genomic instability remained non-significant, with the exception of the effects of age on the number of structural variants (Table 2).

**Table 2:** Mixed effects models predicting the levels of six different measures of instability using the log number of SBS2 and SBS13 mutations and TP53 mutation status, as well as accounting for the effects of tumour type as a random variable. The number of SBS2 and SBS13 mutations was log transformed using the natural logarithm. These models correspond to models 10-15, detailed in Additional file 1: Supplementary Note 1. *SV* structural variant, *CN* copy number, *PGA* Proportion of the Genome Altered, *INDELs* Insertions and Deletions, *ID8* INDEL signature 8, *ID6* INDEL signature 6, *LMM* linear mixed effects model, *NB* negative binomial, *ZINB* zero inflated negative binomial, *AIC* Akaike Information Criterion

	*Dependent variable:*						
		PGA	CN Segments	SVs	INDELs	ID8	ID6
	Model Type	LMM	NB	NB	NB	ZINB	ZINB
Count model: (Intercept)	Coefficient	5.37	4.44	0.257	3.16	0.581	1.62
	95% CI	(−7.32,18.1)	(4.03,4.84)	(−1.36,1.87)	(2.67,3.65)	(−1.44;2.61)	(0.355,2.88)
	*p* value	0.408	1.16×10^−100∗∗∗^	0.755	3.04×10^−41∗∗∗^	0.574	0.0121^∗^
Count model: Age	Coefficient	0.0490	−0.000657	0.0281	0.00315	0.0235	−0.00354
	95% CI	(−0.084,0.182)	(−0.00398,0.00267)	(0.00250,0.0537)	(−0.00049,0.00679)	(−0.00840,0.0554)	(−0.0130,0.00596)
	*p* value	0.472	0.699	0.0314^∗^	0.0903	0.149	0.465
Count model:	Coefficient	5.83	0.241	0.704	0.449	0.575	0.577
log(SBS2/13)	95% CI	(4.34,7.32)	(0.200,0.282)	(0.468,0.940)	(0.391,0.507)	(0.268,0.882)	(0.419,0.735)
	*p* value	7.55×10^−14∗∗∗^	1.20×10^−30∗∗∗^	5.00×10^−9∗∗∗^	3.03×10^−45∗∗∗^	2.44×10^−4∗∗∗^	8.07×10^−13∗∗∗^
Count model: TP53	Coefficient	11.0	0.131	1.26	1.08	0.123	2.16
	95% CI	(7.15,14.9)	(0.039,0.224)	(0.502,2.02)	(0.502,1.65)	(−0.024,0.271)	(0.258,4.07)
	*p* value	3.70×10^−8∗∗∗^	5.27×10^−3∗∗^	0.00114^∗∗^	2.37×10^−4∗∗∗^	0.102	0.0260^∗^
Count model:	Coefficient			−0.156	−0.158		−0.347
log(SBS2/13):TP53	95% CI			(−0.268,−0.045)	(−0.241,−0.0742)		(−0.622,−0.0729)
	*p* value			0.00610^∗∗^	2.17×10^−4∗∗∗^		0.0131^∗^
Count model:	Coefficient			-0.00462		−0.00354	
Age:log(SBS2/13)	95% CI			(-0.00845,-0.000799)		(−0.00834,0.00126)	
	*p* value			0.0178^∗^		0.148	
Zero model: (Intercept)	Coefficient					−0.684	1.07
	95% CI					(−0.839,−0.530)	(0.902,1.24)
	*p* value					4.19×10^−18∗∗∗^	4.23×10^−36∗∗∗^
AIC		6710.3	10,058.3	8687.9	10448.8	6356.6	3266.8
Log likelihood		−3349.1	−5023.2	−4335.9	−5217.4	−3170.3	−1625.4
Num. obs.		725	725	717	725	725	725
Num. tumour types		26	26	26	26	26	26

^∗∗∗^*p*<0.001; ^∗∗^*p*<0.01; ^∗^*p*<0.05

We also investigated the effect of TP53 mutation and APOBEC3 mutations on overall survival by constructing Cox proportional hazards models combined with mixed effects models, taking the effects of tumour type into account (CoxME models). When presence of APOBEC3 mutations is considered alone it does not have a significant effect on survival (*p* = 0.129, hazard ratio = 1.18; Additional file 1: Supplementary table 7). However, when we include TP53 mutation status we find that APOBEC3 mutations increase the hazard ratio when TP53 is not mutated, and TP53 mutation significantly increases the hazard ratio when APOBEC3 mutations are not present, negatively affecting survival in both cases (APOBEC3 mutation presence *p* = 0.0128, hazard ratio = 1.44; TP53 mutation *p* = 0.00318, hazard ratio = 1.47; Additional file 1: Supplementary table 8). The interaction between APOBEC3 mutation presence and TP53 mutation was also significant (*p* = 0.0477, hazard ratio = 0.697), but had a hazard ratio below 1, suggesting that the co-occurrence of APOBEC3 mutations and TP53 mutation result in better survival outcomes.

### The number of non-kataegis APOBEC3 mutations is associated with increased genomic instability

To address whether the results of our models could be attributed to processes such as kataegis, in which APOBECs act on single stranded DNA byproducts of DNA damage repair rather than causing strand breaks themselves, we reconstructed our models excluding SNVs attributed to kataegis events involving APOBEC3 mutations (described in [19]). Excluding APOBEC3 mutations associated with kataegis did not appreciably alter our conclusions. We found that the number of APOBEC3 mutations, excluding those attributed to kataegis, remained a significant predictor for each of our measures of genomic instability when the effects of TP53 mutation were accounted for (Additional file 1: Supplementary Tables 9 and 10). This strongly suggests that APOBECs may play an active role in the generation of widespread and diverse genomic instability.

## Discussion

We show, for the first time using whole genome sequencing data from 24 different tumour types, that increases in APOBEC3 signatures are associated not only with increased mutation burden, but also that the presence, and amount of these mutations correlate with multiple measures of genomic instability across multiple different cancer types. We expand on previous work in the field, which primarily used mutation burden and mutation clusters as measures of genomic instability (see [16] and [27]), and introduce six measures of genomic instability, two of which (INDEL signatures ID6 and ID8) have not been studied before. It has previously been suggested that the increase in base substitutions observed in cancers over-expressing APOBEC3B (A3B) may be due to A3B induced U/G mis-pairs being processed by repair enzymes, which may result in other patterns of mutations, as well as strand breaks and chromosomal rearrangements [16, 28]. Our analysis of the relationship between APOBEC3 mutations and our measures of genomic instability strongly suggests that this is the case and that APOBECs play an active role in the generation of genomic instability.

We found higher levels of structural variants, copy number segments, and INDELs in tumours carrying APOBEC3 mutations (Fig. 2), all common outcomes of double strand break (DSB) repair [29]. In addition, INDEL signatures ID6 and ID8, which have been proposed as indicators of non-homologous end-joining (NHEJ) repair of DSBs, are also present in higher numbers in tumours carrying APOBEC3 mutations [23]. While PGA may not be directly related to DSBs, it may reveal samples in which relatively few but large copy number events may have occurred, as a result of DSBs, which may not necessarily be reflected by the number of copy number segments. Tumours containing APOBEC3 mutations were also found to have higher levels of PGA. The observation that the number of APOBEC3 mutations served as a significant positive predictor for all of the measures of genomic instability, after accounting for variation between tumour types and the effect of TP53 mutation, suggests that the two are closely related.

It can be argued that higher levels of APOBEC3 mutations are a consequence, rather than a cause, of increasing genomic instability. The conventional view of the involvement of APOBEC3 in genomic instability presents APOBEC3 as reactionary to double strand breaks and other processes that result in the generation of single stranded DNA. Several groups have demonstrated the occurrence of clusters of classical APOBEC3 mutations in the vicinity of double strand breaks [12, 27].

However, the immunoglobulin translocations caused by activation induced cytidine deaminase (AID) in B cell tumours serve as a precedent for the generation of DSBs, and their downstream consequences, by cytidine deaminases [18]. AID, which is ancestral to the APOBEC3 enzymes [30], deaminates cytosines in the switch region near the immunoglobulin locus. The resulting uracils are excised by uracil N glycosylase (UNG), resulting in an abasic site which is processed into a single strand break (reviewed in [31]). These single strand breaks can then form double strand breaks, either through further processing of the site, or due to close proximity of multiple single strand breaks [31]. The resolution of the DSBs precipitated by AID in these regions, is the basis of class switch recombination (CSR) [31]. In addition to its role in CSR, off-target activity of AID is known to result in translocations between IGH and various genes, most notably MYC, BCL1, BCL2, MALT1, E2A, and CRLF2 [32]. AID mediated translocations are thought to account for half of all human haematopoietic malignancies [32].

APOBEC3 can undoubtedly be activated in response to, and act on, the products of DNA damage. Our results suggest that it can also be a contributing factor in DNA damage and genomic instability. Kataegis is associated with so-called ‘opportunistic’ action of APOBECs at single stranded DNA during repair of DNA strand breaks. When we exclude mutations attributed to kataegis from our analysis, the strong association between APOBEC3 mutations and genomic instability remains in place for five of the six measures of genomic instability that we investigated. Thus, our results support a model of APOBEC3 mediated mutagenesis resulting in genomic instability via double strand break formation, which we posit mirrors the effects of AID in B cell tumours.

Associations between APOBEC3 signature prevalence and genomic instability were observed across multiple tumour types. Particularly strong correlations were seen for pancreatic cancer, pancreatic endocrine neoplasms, kidney renal clear cell carcinoma, kidney renal papillary cell carcinoma, malignant lymphoma, bone cancer, and uterine corpus endometrial carcinoma (Fig. 3).

Although large studies of pancreatic cancer genomes have highlighted APOBEC3 activity as one of the main mutagenic processes in pancreatic cancer [33–35], the role of APOBEC3 activity in pancreatic cancer appears not to have been studied in great detail. However, preliminary data suggest that APOBEC3A activity may result in widespread genomic instability through a non-deaminase dependent mechanism, in a mouse model of pancreatic cancer [36], suggesting the possibility of novel therapeutics for pancreatic cancer.

The presence of APOBEC3 related mutations in kidney cancer has also not been studied in great detail. Although we observe significantly higher levels of genomic instability in both kidney renal clear cell carcinoma and kidney renal papillary cell carcinomas that carry APOBEC3 mutations, we urge caution when interpreting these results, as they are based on relatively few positive samples (2 and 3 positive samples, respectively). Further work is required to completely understand the role that APOBEC3 mediated mutagenesis may play in kidney cancer.

Interestingly, bone cancer and APOBEC3 induced genomic instability have been linked through the presence of kataegis in 50–85% of osteosarcoma samples [37, 38]. In addition to kataegis, osteosarcomas frequently display high levels of genomic instability, in the form of structural rearrangements and copy number aberrations, as well as carrying mutations in TP53 [37, 38]. It would be interesting to see if any of these abnormalities may be linked to the activity of APOBEC3 enzymes.

Our analysis of TP53 mutations in this data set lends further support to work by other groups, in which TP53 mutations are observed more frequently in tumours expressing high levels of APOBEC3B [16]. TP53 mutation has previously been linked with aneuploidy and copy number variations [39], and in this study positively associated with the number of copy number segments, PGA, structural variants, INDELs, and ID6 INDELs. Despite the inclusion of TP53 status, the number of APOBEC3 mutations was consistently identified as a highly significant predictor for all six measures of genomic instability.

We found that both the presence of APOBEC3 mutations, and missense or nonsense mutations in TP53 each had a negative effect on survival, but conferred a survival advantage when they occurred together. It has been suggested that cancers with an APOBEC3 mutation component could be treated with DNA damaging drugs, resulting in synthetic lethality [11]. This is an interesting idea, and evidence from studies of urothelial carcinoma suggests that this may indeed improve treatment outcomes [13, 14]. Similarly, it has recently been reported that a subset of clear cell ovarian carcinoma (CCOC) patients over-expressing A3B had better survival outcomes when treated with platinum based drugs [15]. It was theorised that the increased survival of the patients in this CCOC subset was due to A3B mediated DNA damage sensitising the tumour cells to further damage by platinum based drugs [15]. This suggests that A3B activity and the presence of APOBEC3 related mutations may be used to inform treatment decisions and may also provide an insight into treatment outcomes [13, 15]. Our results suggest that this approach may be beneficial for patients with pancreatic cancer, kidney cancer, malignant lymphoma, bone cancer, and uterine corpus endometrial carcinoma, carrying APOBEC3 mutations.

## Conclusions

In this study we investigate the relationship between the presence of mutational signatures attributed to the APOBEC3 family of cytidine deaminases and panel of measures of genomic instability. Using a series of mixed effects models we demonstrate that APOBEC3 mutations are associated with increased mutation burden, SVs, copy number segments, INDELs, and ID8 INDELs. Furthermore, this relationship holds when the presence of TP53 mutations is accounted for, as well as when mutations attributed to kataegis are excluded from the analysis.

Our data suggest that, in addition to being responsible for genomic instability in the form of clustered mutations (kataegis), APOBEC3 deaminases may also play a causative role in the generation of genomic instability, analogous to the effects of AID in haematopoietic malignancies. In particular, the association between APOBEC3 mutations and the number of ID8 indels, which are attributed to NHEJ of DSBs, the number of SVs, and the number of copy number segments suggests that APOBEC3s may be involved in the generation of DSBs.

## Methods

### Data

In this study we analysed whole genome sequencing of 2451 white listed primary tumour samples made available through the Pan-Cancer Analysis of Whole Genomes (PCAWG) consortium [19]. The full data set consists of 2600 samples, however, we restricted our analysis to primary tumours included on PCAWG’s white list. PCAWG data can be accessed through the ICGC at http://dcc.icgc.org/pcawg/. Access to controlled data was granted by the International Cancer Genome Consortium (ICGC) Data Access Compliance Office (DACO) for the ICGC portion of the PCAWG data, and by The Cancer Genome Atlas (TCGA) Data Access Committee for the TCGA portion of the data.

Analysis of the mutational signatures was carried out by the PCAWG Mutation Signatures and Processes working group [23]. For the analysis reported in this paper we used signatures called using SigProfiler. We also made use of structural variation data, which was made available through the PCAWG Structural Variation working group [40]. Clustered mutation data related to kataegis was provided by the Evolution and Heterogeneity working group [19].

Of the 2451 white listed samples, 741 carried mutations attributed to SBS2 and SBS13. These 741 samples were used for calculating the correlation between APOBEC3 SNVs and non-APOBEC3 SNVs.

### Mixed Effects Models

Mixed effects models were created using version 1.1–23 of the ‘lme4’ R package and version 1.0.2.1 of ‘glmmTMB’ R package [41, 42]. The results of the linear and mixed effects models were presented using version 5.2.2 of the ‘Stargazer’ R package and version 1.37.5 of the ‘texreg’ R package [43, 44]. A full list of models can be found in Additional file 1: Supplementary Note 1.

We created three mixed effects models to account for the effect of tumour type on the relationship between the number of APOBEC3 mutations, age, and the two combined on the total number of non-APOBEC3 mutations (Additional file 1: Supplementary Note 1, equations 1–3, *n* = 725, 741, and 725, respectively). In addition, six mixed effects models were created to investigate the relationship between the number of APOBEC3 mutations, and the six measures of genomic instability that we investigated (Additional file 1: Supplementary Note 1, equations 4–9, *n* = 725 for models of PGA, CN segments, INDELs, ID8, and ID6. *n* = 717 for models of SVs). A further six models were constructed to investigate the additional effect of TP53 mutation (Additional file 1: Supplementary Note 1, equations 10–15, *n* = 725 for models of PGA, CN segments, INDELs, ID8, and ID6. *n* = 717 for models of SVs). Models in which we exclude mutations attributed to kataegis were constructed using the same formulas as models 4-15 (Additional file 1: Supplementary Note 1, Additional file 1: Supplementary tables 9 and 10, *n* = 724 for models of PGA, CN segments, INDELs, ID6, and ID8. *n* = 716 for models of SVs. *n* = 724 for models of CN segments, INDELs, ID6, and ID8 accounting for TP53 mutation. *n* = 678 for models of PGA accounting for TP53 mutation. *n* = 706 for models of SVs accounting for TP53 mutation).

For mixed effects modelling of the relationship between number of APOBEC3 mutations and genomic instability we only consider samples which contain APOBEC3 mutations. The number of mutations located in kataegis clusters attributed to APOBEC3 were subtracted from the total number of SBS2 and SBS13 mutations; samples for which this produced a negative number of mutations were excluded from our analysis.

For each measure of genomic instability we formulated models with and without interaction terms between the dependent variables that were surveyed. We also built models based on different distributions for the independent variable (e.g. the normal distribution, negative binomial distribution, and the negative binomial distribution). We selected the optimum model for each measure by selecting the model with the lowest Akaike information criterion (AIC) and a *p* value <0.05 when compared to other models using an ANOVA.

### Survival analysis

Survival analysis and generation of Cox Proportional Hazard mixed effects models was carried out using the ‘survminer’, ‘survival’, and ‘coxme’ packages for R [21, 45, 46]. The patient’s overall survival was used as an endpoint. The CoxME models generated are described in detail in equations 19 and 20 of Additional file 1: Supplementary Note 1 (*n* = 1492).

### Genomic instability

Genomic instability is characterised by a range of different changes at the chromosome level. Frequent changes include increased numbers of insertions, deletions, translocations, and structural variants [47]. We were able to assess the number of each of these changes using data provided by the PCAWG Structural Variation working group [40].

Changes in ploidy have also been associated with genomic instability [47]. We assessed changes in ploidy by investigating the proportion of the genome altered (PGA), which describes the proportion of the genome that deviates from copy number 2 or 4, for diploid and whole genome duplicated samples, respectively. We also examined the number of copy number segments, which provides an insight into the number of copy number changes across the genome.

In addition, we assessed the number of insertions and deletions (INDELS) that are attributed to INDEL signatures ID6 and ID8. Both ID6 and ID8 have been attributed to error prone non-homologous end-joining repair of double strand breaks [23]. Double strand breaks, when repaired incorrectly, can lead to translocations and genomic instability [48]. We reasoned that increased numbers of DNA breaks caused by increased APOBEC3 activity could also be detected as increased levels of ID6 and ID8, reflecting elevated DNA damage repair activity, as well as higher numbers of translocations and INDELs as outcome measures.

### Volcano plot

To aid with visualisation, and to prevent division by 0 when estimating effect sizes, a pseudocount of 1 was added to the medians of the genome instability measures calculated for tumours in each tumour type that either carry SBS2 and SBS13 mutations or do not carry these mutations, with the exception of PGA, before the ratio of the medians was taken. All statistical analysis was carried out on the raw data, without a pseudocount. The number of samples used in this analysis is represented in Additional file 1: Supplementary Table 1 (*n* = 2451 total). Details of the means and median ratios, and *p* values for each of the tumour type and genomic instability measure combinations are presented in Additional file 2: Supplementary Data 1.

## Supplementary Information

**Additional file 1** Supplementary Tables 1-10, Supplementary Note 1, Supplementary Note 2. Supplementary Table 1 - Spearman correlation coefficients for the correlation between log number ofAPOBEC mutations and the log number of non-APOBEC mutations by tumour type. Supplementary Table 2 - Mixed Effects Models for Predicting the Number of non-APOBEC3 mutations. Supplementary Table 3 - Wilcoxon rank sum test, comparing the levels of each of the genomic instability measures between samples containing SBS2 and SBS13 mutations, and those not containing SBS2 and SBS13 mutations Supplementary Table 4 - The number of samples belonging to each tumour type that either contain SBS2 and SBS13 mutations, or do not contain SBS2 and SBS13 mutations. Supplementary Table 5 - Combined *p*-values using Fisher’s combined probability test. Supplementary Table 6 - Akaike Information Criteria (AIC) and *p*-values from ANOVAs comparing models with and without TP53 status for each GI measures. Supplementary Table 7 - Coxme model using only SBS2 and SBS13 presence to predict survival. Supplementary Table 8 - Coxme model using SBS2 and SBS13 presence, p53 mutation status, and the interaction between them as predictors of survival. Supplementary Table 9 - Mixed effects models predicting the levels of six different measures of instability using age, the number of SBS2 and SBS13 mutations excluding those attributed to kataegis, accounting for the effects of tumour type as a random variable. Supplementary Table 10 - Mixed Effects Models Predicting the levels of six different measures of instability using the log number of SBS2 and SBS13 mutations excluding those attributed to kataegis, and TP53 mutation status, as well as accounting for the effects of tumour type as a random variable. Supplementary Note 1 - List of mixed effects models. Supplementary Note 2 - A note on Fisher *p*-value combinations.

**Additional file 2** Supplementary Data 1. Excel file containing details of the means and median ratios, and *p*-values for each of the tumour type and genomic instability measure combinations described in Fig. 3.

## Data Availability

The data sets generated and/or analysed during the current study are available to download from https://dcc.icgc.org/releases/PCAWG.

## Abbreviations

AID: Activation induced cytidine deaminase
APOBEC3: Apolipoprotein B mRNA editing enzyme catalytic polypeptide 3
CN: Copy number
CNA: Copy number aberration
DSB: Double strand breaks
ICGC: International Cancer Genome Consortium
ID: INDEL signature
INDELs: Insertions and deletions
NHEJ: Non-homologous end-joining
PCAWG: Pan-Cancer Analysis of Whole Genomes
PGA: Proportion of the genome altered
SBS: Single base substitution
SV: Structural variant
TCGA: The Cancer Genome Atlas
