# Supplementary material for: APOBEC3 mutational signatures are associated with extensive and diverse genomic instability across multiple tumour types
Source: BMC Biol. 2022 May 21;20:117. doi: 10.1186/s12915-022-01316-0 (PMC9124393; doi:10.1186/s12915-022-01316-0)
Supplement: Supplementary file 1 — Additional file 1 Supplementary Tables 1-10, Supplementary Note 1, Supplementary Note 2. Supplementary Table 1 - Spearman correlation coefficients for the correlation between log number ofAPOBEC mutations and the log number of non-APOBEC mutations by tumour type. Supplementary Table 2 - Mixed Effects Models for Predicting the Number of non-APOBEC3 mutations. Supplementary Table 3 - Wilcoxon rank sum test, comparing the levels of each of the genomic instability measures between samples containing SBS2 and SBS13 mutations, and those not containing SBS2 and SBS13 mutations Supplementary Table 4 - The number of samples belonging to each tumour type that either contain SBS2 and SBS13 mutations, or do not contain SBS2 and SBS13 mutations. Supplementary Table 5 - Combined p-values using Fisher’s combined probability test. Supplementary Table 6 - Akaike Information Criteria (AIC) and p-values from ANOVAs comparing models with and without TP53 status for each GI measures. Supplementary Table 7 - Coxme model using only SBS2 and SBS13 presence to predict survival. Supplementary Table 8 - Coxme model using SBS2 and SBS13 presence, p53 mutation status, and the interaction between them as predictors of survival. Supplementary Table 9 - Mixed effects models predicting the levels of six different measures of instability using age, the number of SBS2 and SBS13 mutations excluding those attributed to kataegis, accounting for the effects of tumour type as a random variable. Supplementary Table 10 - Mixed Effects Models Predicting the levels of six different measures of instability using the log number of SBS2 and SBS13 mutations excluding those attributed to kataegis, and TP53 mutation status, as well as accounting for the effects of tumour type as a random variable. Supplementary Note 1 - List of mixed effects models. Supplementary Note 2 - A note on Fisher p-value combinations. [file 12915_2022_1316_MOESM1_ESM.pdf]

Supplementary Material

Supplementary Tables

Supplementary Table 1: The number of samples belonging to each tumour type that either contain SBS2 and SBS13 mutations, or do not contain SBS2 and SBS13 mutations.

	Tumour type	Number of samples with SBS2 and SBS13 mutations	Number of samples without SBS2 and SBS13 mutations	Total number of samples
1	BLCA	23	0	23
2	BOCA	22	39	61
3	BRCA	185	24	209
4	BTCA	9	3	12
5	CESC	18	2	20
6	CLLE	0	90	90
7	CMDI	0	30	30
8	COAD	0	37	37
9	DLBC	2	5	7
10	EOPC	0	41	41
11	ESAD	32	65	97
12	GACA	14	18	32
13	GBM	0	30	30
14	HNSC	43	0	43
15	KICH	9	34	43
16	KIRC	2	35	37
17	KIRP	3	29	32
18	LAML	0	8	8
19	LGG	0	18	18
20	LICA	0	5	5
21	LIHC	0	53	53
22	LINC	0	28	28
23	LIRI	5	245	250
24	LUAD	22	15	37
25	LUSC	39	8	47
26	MALY	7	93	100
27	ORCA	10	3	13
28	OV	49	52	101
29	PACA	145	85	230
30	PAEN	27	54	81
31	PBCA	0	230	230
32	PRAD	2	151	153
33	READ	0	15	15
34	RECA	1	73	74
35	SARC	24	10	34
36	SKCM	0	2	2
37	STAD	18	18	36
38	THCA	10	38	48
39	UCEC	20	24	44

Supplementary Table 2: Spearman correlation coefficients for the correlation between log number of APOBEC mutations and the log number of non-APOBEC mutations by tumour type. p-values were corrected for multiple testing using the Benjamini-Hochberg procedure for controlling false discovery rate (FDR) at $\alpha = 0.05$.

Project Code	Project Name	n	ρ	p-value	Significance	Adjusted p-value
BLCA	Bladder Urothelial Carcinoma	23	0.161	0.462	Not significant	0.535
BOCA	Bone Cancer	22	0.917	1.9×10^{-9}	Significant	1.05×10^{-8}
BRCA	Breast Cancer	185	0.247	6.87×10^{-4}	Significant	0.00151
BTCA	Biliary Tract Cancer	9	0.225	0.56	Not significant	0.616
CESC	Cervical Squamous Cell Carcinoma	18	0.132	0.602	Not significant	0.63
DLBC	Diffuse Large B-Cell Lymphoma	2				
ESAD	Esophageal Adenocarcinoma	32	0.881	2.8×10^{-11}	Significant	3.08×10^{-10}
GACA	Gastric Cancer	14	0.986	9.97×10^{-11}	Significant	7.31×10^{-10}
HNSC	Head and Neck Squamous Cell Carcinoma	43	0.535	2.21×10^{-4}	Significant	6.09×10^{-4}
KICH	Kidney Chromophobe	9	0.921	4.23×10^{-4}	Significant	0.00103
KIRC	Kidney Renal Clear Cell Carcinoma	2				
KIRP	Kidney Renal Papillary Cell Carcinoma	3	-0.247	0.841	Not significant	0.841
LIRI	Liver Cancer	5	0.883	0.0472	Not significant	0.0649
LUAD	Lung Adenocarcinoma	22	0.801	7.58×10^{-6}	Significant	2.78×10^{-5}
LUSC	Lung Squamous Cell Carcinoma	39	0.328	0.0413	Not significant	0.0606
MALY	Malignant Lymphoma	7	0.8	0.0308	Significant	0.0483
ORCA	Oral Cancer	10	0.84	0.00234	Significant	0.00468
OV	Ovarian Cancer	49	0.603	4.62×10^{-6}	Significant	2.03×10^{-5}
PACA	Pancreatic Cancer	145	0.676	1.08×10^{-20}	Significant	2.38×10^{-19}
PAEN	Pancreatic Cancer Endocrine Neoplasms	27	0.514	0.00615	Significant	0.0113
PRAD	Prostate Cancer of Adenocarcinoma	2				
RECA	Renal Clear Cell Carcinoma	1				
SARC	Sarcoma	24	0.528	0.00794	Significant	0.0134
STAD	Gastric Adenocarcinoma	18	0.834	1.66×10^{-5}	Significant	5.22×10^{-5}
THCA	Thyroid Cancer	10	0.354	0.316	Not significant	0.386
UCEC	Uterine Corpus Endometrial Carcinoma	20	0.299	0.201	Not significant	0.26

Supplementary Table 3: Mixed Effects Models for Predicting the Number of non-APOBEC3 mutations. Models 1, 2, and 3 correspond to models 1, 2, and 3 described in Supplementary Note 1. Model 1 only considers the effects of age on the total mutation burden excluding APOBEC3 mutations. Model 2 considers only the effects of APOBEC3 mutations, and model 3 considers both the effects of age and the number of APOBEC3 mutations on the non-APOBEC3 mutation burden.

	Dependent variable:		
	log(Number of non-APOBEC3 mutations)		
	(1)	(2)	(3)
Age	Coefficient: 0.010<br>p = 3.76 x 10^{-6} ***<br>95% CI (0.006, 0.014)		Coefficient: 0.007<br>p = 2.26 x 10^{-3} **<br>95% CI (0.003, 0.010)
log(Number of APOBEC3 mutations)		Coefficient: 0.342<br>p = 4.16 x 10^{-56} ***<br>95% CI (0.303, 0.381)	Coefficient: 0.349<br>p = 2.27 x 10^{-49} ***<br>95% CI (0.310, 0.388)
Constant	Coefficient: 8.12<br>p = 3.52 x 10^{-51} ***<br>95% CI (7.746, 8.499)	Coefficient: 6.415<br>p = 1.08 x 10^{-60} ***<br>95% CI (6.061, 6.770)	Coefficient: 5.996<br>p = 2.45 x 10^{-63} ***<br>95% CI (5.608, 6.384)
Observations	725	741	725
Log Likelihood	−831.379	−741.003	−703.723
Akaike Inf. Crit.	1670.759	1490.005	1417.445
Bayesian Inf. Crit.	1689.104	1508.437	1440.376
Note:			*p<0.05; **p<0.01; ***p<0.001

Supplementary Table 4: Wilcoxon rank sum test, comparing the levels of each of the genomic instability measures between samples containing SBS2 and SBS13 mutations, and those not containing SBS2 and SBS13 mutations. Results are represented visually in Figure 2. n = 2,451 for INDELs, ID8, ID6, PGA and Copy Number Segments. n = 2,427 for SVs.

Measure	*p*-value	Significance	Adjusted *p*-value
SVs	8.71×10^{-100}	Significant	5.22×10^{-99}
Copy Number Segments	3.56×10^{-90}	Significant	1.07×10^{-89}
PGA	1.15×10^{-86}	Significant	2.30×10^{-86}
INDELs	7.71×10^{-15}	Significant	7.71×10^{-15}
ID8	4.01×10^{-20}	Significant	4.81×10^{-20}
ID6	7.74×10^{-34}	Significant	1.16×10^{-33}

Supplementary Table 5: Combined p-values using Fisher's combined probability test. p-values were combined for the different measures of genomic instability within each tumour type, and the resulting Fisher method p-values were corrected for multiple testing using the Benjamini-Hochberg procedure for controlling false discovery rate (FDR) at $\alpha = 0.05$.

Tumour Type	Number of GI measures	Fisher method p-value	Significance	Adjusted p-value
BOCA	6	3.14 x 10^{-5}	Significant	9.42 x 10^{-5}
BRCA	6	7.77 x 10^{-8}	Significant	3.73 x 10^{-7}
BTCA	5	9.13 x 10^{-3}	Significant	0.0183
CESC	5	0.0151	Significant	0.0241
DLBC	6	0.975	Not significant	0.975
ESAD	6	0.298	Not significant	0.358
GACA	6	0.202	Not significant	0.262
KICH	6	0.0101	Significant	0.0186
KIRC	6	1.92 x 10^{-4}	Significant	4.19 x 10^{-4}
KIRP	6	3.17 x 10^{-7}	Significant	1.27 x 10^{-6}
LIRI	6	0.0638	Not significant	0.0958
LUAD	6	2.95 x 10^{-5}	Significant	9.423 x 10^{-5}
LUSC	6	0.944	Not significant	0.975
MALY	6	5.76 x 10^{-10}	Significant	4.61 x 10^{-9}
ORCA	6	0.922	Not significant	0.975
OV	6	0.206	Not significant	0.262
PACA	6	3.47 x 10^{-13}	Significant	8.34 x 10^{-12}
PAEN	6	9.75 x 10^{-11}	Significant	1.17 x 10^{-9}
PRAD	6	7.29 x 10^{-5}	Significant	1.94 x 10^{-4}
RECA	6	0.207	Not significant	0.262
SARC	5	1.03 x 10^{-4}	Significant	0.000247
STAD	6	0.0130	Significant	0.0223
THCA	5	0.545	Not significant	0.623
UCEC	6	3.67 x 10^{-9}	Significant	2.20 x 10^{-8}

Supplementary Table 6: Akaike Information Criteria (AIC) and p-values from ANOVAs comparing models with and without TP53 status for each GI measures. Full models are specified in Supplementary Note 1.

GI Measure	p-value	AIC without TP53	AIC with TP53
PGA	4.02×10^{-8}	6738	6710
Copy Number Segments	5.32×10^{-3}	10064	10058
Structural Variants	1.75×10^{-4}	8701	8687
INDELs	1.00	10444	10448
ID8	0.101	6357	6356
ID6	0.0133	3270	3266

Supplementary Table 7: Coxme model using only SBS2 and SBS13 presence to predict survival (n = 1,492)

**Random Effects**	N	Standard Deviation	Variance		
Tumour Type	36	1.14	1.31		
**Fixed Effects**	Coefficient	Hazard Ratio	95% CI	z	*p*
SBS2/13 presence	0.162	1.18	(0.95,1.45)	1.52	0.127

Supplementary Table 8: Coxme model using SBS2 and SBS13 presence, p53 mutation status, and the interaction between them as predictors of survival. (n = 1,492)

**Random Effects**	N	Standard Deviation	Variance		
Tumour Type	36	1.14	1.31		
**Fixed Effects**	Coefficient	Hazard Ratio	95% CI	z	*p*
SBS2/13 Presence	0.366	1.44	(1.08,1.92)	2.49	0.0128*
TP53 Mutation	0.384	1.47	(1.14,1.90)	2.95	0.00318**
SBS2/13 Presence*TP53 Muation	-0.362	0.697	(0.487,0.996)	-1.98	0.0477*

Supplementary Table 9: Mixed effects models predicting the levels of six different measures of instability using age, the number of SBS2 and SBS13 mutations excluding those attributed to kataegis, accounting for the effects of tumour type as a random variable. The number of remaining SBS2 and SBS13 mutations was log transformed using the natural logarithm. These models correspond to models 3-9, detailed in Supplementary Note 1. (SV = Structural Variant, CNV = Copy Number, PGA = Proportion of the Genome Altered, INDELs = Insertions and Deletions, ID8 = INDEL signature 8, ID6 = INDEL signature 6, LMM = Linear Mixed Effects Model, NB = Negative Binomial, ZINB = Zero Inflated Negative Binomial, AIC = Akaike Information Criterion, BIC = Bayesian Information Criterion)

		Dependent variable:					
		PGA	CN Segments	SVs	INDELs	ID8	ID6
	Model Type	LMM	NB	NB	NB	ZINB	ZINB
Count model: (Intercept)	Coefficient	7.67	4.72	1.33	1.65	0.233	5.19
	95% CI	(−5.05, 20.4)	(4.31, 5.13)	(−0.220, 2.88)	(0.613, 2.68)	(−1.54, 2.01)	(1.18, 9.21)
	p-value	0.238	$3.38 \times 10^{-114***}$	0.0926	$1.79 \times 10^{-03**}$	0.796	0.0112^{*}
Count model: Age	Coefficient	0.0687	$-2.37x10^{-7}$	0.0265	0.0381	0.0319	−0.0522
	95% CI	(−0.0684, 0.206)	(−0.00341, 0.00341)	(0.00257, 0.0503)	(0.0219, 0.0542)	(0.00426, 0.0596)	(−0.114, 0.00976)
	p-value	0.326	1.00	0.0299^{*}	$3.95 \times 10^{-6***}$	0.024^{*}	0.0987
Count model: log(SBS2/13 - kataegis)	Coefficient	5.97	0.204	0.554	0.696	0.645	0.056
	95% CI	(4.52, 7.42)	(0.165, 0.244)	(0.324, 0.783)	(0.540, 0.852)	(0.370, 0.920)	(−0.523, 0.636)
	p-value	$3.67 \times 10^{-15***}$	$2.35 \times 10^{-24***}$	$2.21 \times 10^{-6***}$	$2.83 \times 10^{-9***}$	$4.19 \times 10^{-6***}$	0.849
Count model: Age: log(SBS2/13 - kataegis)	Coefficient			-0.00428	−0.00545	−0.00488	0.00686
	95% CI			(-0.00791,-0.000651)	(−0.00790, −0.00299)	(−0.00914, −0.000621)	(-0.0021,0.0158)
	p-value			0.0208^{*}	$1.41 \times 10^{-5***}$	0.0247^{*}	0.134
Zero model: (Intercept)	Coefficient					−0.682	1.07
	95% CI					(−0.837, −0.528)	(0.901, 1.23)
	p-value					$5.40 \times 10^{-18***}$	$5.42 \times 10^{-36***}$
AIC		6735.3	10081.1	8722.8	10430.9	6339.6	3267.7
BIC		6758.2	10104.0	8750.2	10458.4	6371.7	3299.8
Log Likelihood		−3362.6	−5035.6	−4355.4	−5209.5	−3162.8	−1626.9
Num. obs.		724	724	716	724	724	724
Num. Tumour Types		26	26	26	26	26	26

$^{***}p < 0.001$; $^{**}p < 0.01$; $^{*}p < 0.05$

Supplementary Table 10: Mixed Effects Models Predicting the levels of six different measures of instability using the log number of SBS2 and SBS13 mutations excluding those attributed to kataegis, and TP53 mutation status, as well as accounting for the effects of tumour type as a random variable. The number of remaining SBS2 and SBS13 mutations was log transformed using the natural logarithm. These models correspond to models 10-15, detailed in Supplementary Note 1. (SV = Structural Variant, CNV = Copy Number Variation, PGA = Proportion of the Genome Altered, INDELs = Insertions and Deletions, ID8 = INDEL signature 8, ID6 = INDEL signature 6, LMM = Linear Mixed Effects Model, NB = Negative Binomial, ZINB = Zero Inflated Negative Binomial, AIC = Akaike Information Criterion, BIC = Bayesian Information Criterion)

		Dependent variable:					
		PGA	CN Segments	SVs	INDELs	ID8	ID6
	Model Type	LMM	NB	NB	NB	ZINB	ZINB
(Intercept)	Coefficient	8.97	4.73	1.23	3.42	0.313	1.60
	95% CI	(−3.32, 21.3)	(4.32, 5.13)	(−0.292, 2.75)	(2.96, 3.89)	(−1.46, 2.09)	(0.362, 2.83)
	p-value	0.154	$2.29\text{x}10^{-115***}$	0.113	$3.16\text{x}10^{-47***}$	0.729	0.0113^{*}
Count model: Age	Coefficient	0.0536	−0.000350	0.0214	0.00310	0.0301	−0.00366
	95% CI	(−0.0803, 0.188)	(−0.00375, 0.00305)	(−0.00234, 0.0452)	(−0.000557, 0.00676)	(0.00234, 0.0578)	(−0.0131, 0.00574)
	p-value	0.433	0.840	0.0772	0.0966	0.0335^{*}	0.445
Count model: log(SBS2/13 - kataegis)	Coefficient	5.35	0.199	0.568	0.419	0.628	0.584
	95% CI	(3.93, 6.78)	(0.160, 0.239)	(0.343, 0.793)	(0.365, 0.473)	(0.353, 0.903)	(0.430, 0.737)
	p-value	$6.78\text{x}10^{-13***}$	$1.73\text{x}10^{-23***}$	$7.64\text{x}10^{-7***}$	$8.25\text{x}10^{-52***}$	$7.49\text{x}10^{-6***}$	$9.32\text{x}10^{-14***}$
Count model: TP53	Coefficient	11.2	0.148	1.13	0.957	0.130	2.20
	95% CI	(7.34, 15.1)	(0.0540, 0.242)	(0.449, 1.82)	(0.440, 1.47)	(−0.0172, 0.277)	(0.353, 4.05)
	p-value	$2.27\text{x}10^{-8***}$	$2.02\text{x}10^{-3**}$	$1.17\text{x}10^{-3**}$	$2.85\text{x}10^{-4***}$	0.0835	0.0196^{*}
Count model: log(SBS2/13 - kataegis):TP53	Coefficient			−0.136	−0.141		−0.356
	95% CI			(−0.238, −0.0344)	(−0.218, −0.0650)		(−0.624, −0.0870)
	p-value			$8.73\text{x}10^{-3**}$	$2.89\text{x}10^{-4***}$		$9.48\text{x}10^{-3**}$
Count model: Age: log(SBS2/13 - kataegis)	Coefficient			-0.00365		−0.00464	
	95% CI			(-0.00726,-0.0000348)		(−0.00890, −0.000380)	
	p-value			0.0478^{*}		0.0328^{*}	
Zero model: (Intercept)	Coefficient					−0.682	1.07
	95% CI					(−0.837, −0.528)	(0.900, 1.23)
	p-value					$5.39\text{x}10^{-18***}$	$6.03\text{x}10^{-36***}$
AIC		6706.2	10073.6	8708.5	10437.4	6338.6	3262.9
BIC		6733.7	10101.1	8745.0	10469.5	6375.2	3299.6
Log Likelihood		−3347.1	−5030.8	−4346.2	−5211.7	−3161.3	−1623.4
Num. obs.		678	724	716	724	724	724
Num. groups: dcc_project_4		26	26	26	26	26	26

$^{***}p < 0.001$; $^{**}p < 0.01$; $^{*}p < 0.05$

Supplementary Notes

Supplementary Note 1: List of Mixed Effects Models

The models discussed in the paper are listed below in the lme4 and glmmTMB fomula syntax. We have tried to be consistent in how we constructed our models, however, they do differ with regard to the distribution used to model the independent variable, and the structure of the fixed effects (e.g. some models include interaction terms between different fixed effects, whereas others do not). Ultimately, we decided to choose the model that reflected the data in the most appropriate way. The criteria that we used for model selection consisted of choosing the model with the lowest Akaike Information Criterion value, as well as comparing different models using an ANOVA, and selecting the one performed significantly better than the others.

We acknowledge that our models do not paint a complete picture of the cellular processes contributing to genomic instability, but they do provide a valuable insight into the effects of APOBEC on genomic instability and mutation burden.

Models of Mutation Burden

$$\begin{gathered} log(\text{non-SBS2/SBS13}) \sim \text{Age} + (1|\text{Tumour Type}) \\ \text{Model family: Linear Mixed Effects Model} \\ \text{R package: lme4} \end{gathered} \tag{1}$$

$$\begin{gathered} log(\text{non-SBS2/SBS13}) \sim log(\text{SBS2/SBS13}) + (1|\text{Tumour Type}) \\ \text{Model family: Linear Mixed Effects Model} \\ \text{R package: lme4} \end{gathered} \tag{2}$$

$$\begin{gathered} log(\text{non-SBS2/SBS13}) \sim \text{Age} + log(\text{SBS2/SBS13}) + (1|\text{Tumour Type}) \\ \text{Model family: Linear Mixed Effects Model} \\ \text{R package: lme4} \end{gathered} \tag{3}$$

Models of Genomic Instability

$$\begin{gathered} \text{PGA} \sim \text{Age} + log(\text{SBS2/SBS13}) + (1|\text{Tumour Type}) \\ \text{Model family: Linear Mixed Effects Model} \\ \text{R package: lme4} \end{gathered} \tag{4}$$

$$\begin{gathered} \text{CN Segments} \sim \text{Age} + log(\text{SBS2/SBS13}) + (1|\text{Tumour Type}) \\ \text{Model family: Negative Binomial Mixed Effects Model} \\ \text{R package: glmmTMB} \end{gathered} \tag{5}$$

$$\begin{gathered} \text{SVs} \sim \text{Age} + log(\text{SBS2/SBS13}) + \text{Age} \times log(\text{SBS2/SBS13}) + (1|\text{Tumour Type}) \\ \text{Model family: Negative Binomial Mixed Effects Model} \\ \text{R package: glmmTMB} \end{gathered} \tag{6}$$

$$\begin{gathered} \text{INDELs} \sim \text{Age} + log(\text{SBS2/SBS13}) + \text{Age} \times log(\text{SBS2/SBS13}) + (1|\text{Tumour Type}) \\ \text{Model family: Negative Binomial Mixed Effects Model} \\ \text{R package: glmmTMB} \end{gathered} \tag{7}$$

$$\begin{gathered} \text{ID8} \sim \text{Age} + log(\text{SBS2/SBS13}) + \text{Age} \times log(\text{SBS2/SBS13}) + (1|\text{Tumour Type}) \\ \text{Model family: Zero Inflated Negative Binomial Mixed Effects Model} \\ \text{R package: glmmTMB} \end{gathered} \tag{8}$$

$$\begin{gathered} \text{ID6} \sim \text{Age} + log(\text{SBS2/SBS13}) + \text{Age} \times log(\text{SBS2/SBS13}) + (1|\text{Tumour Type}) \\ \text{Model family: Zero Inflated Negative Binomial Mixed Effects Model} \\ \text{R package: glmmTMB} \end{gathered} \tag{9}$$

Models of Genomic Instability including TP53 Mutation Status

$$\begin{gathered}\text{PGA} \sim \text{Age} + log(\text{SBS2/SBS13}) + \text{TP53} + (1|\text{Tumour Type}) \\ \text{Model family: Linear Mixed Effects Model} \\ \text{R package: lme4}\end{gathered} \tag{10}$$

$$\begin{gathered}\text{CN Segments} \sim \text{Age} + log(\text{SBS2/SBS13}) + \text{TP53} + (1|\text{Tumour Type}) \\ \text{Model family: Negative Binomial Mixed Effects Model} \\ \text{R package: glmmTMB}\end{gathered} \tag{11}$$

$$\begin{gathered}\text{SVs} \sim \text{Age} + log(\text{SBS2/SBS13}) + \text{TP53} + \text{Age} \times log(\text{SBS2/SBS13}) + log(\text{SBS2/SBS13}) \times \text{TP53} + (1|\text{Tumour Type}) \\ \text{Model family: Negative Binomial Mixed Effects Model} \\ \text{R package: glmmTMB}\end{gathered} \tag{12}$$

$$\begin{gathered}\text{INDELs} \sim \text{Age} + log(\text{SBS2/SBS13}) + \text{TP53} + log(\text{SBS2/SBS13}) \times \text{TP53} + (1|\text{Tumour Type}) \\ \text{Model family: Negative Binomial Mixed Effects Model} \\ \text{R package: glmmTMB}\end{gathered} \tag{13}$$

$$\begin{gathered}\text{ID8} \sim \text{Age} + log(\text{SBS2/SBS13}) + \text{Age} \times log(\text{SBS2/SBS13}) + \text{TP53} + (1|\text{Tumour Type}) \\ \text{Model family: Zero Inflated Negative Binomial Mixed Effects Model} \\ \text{R package: glmmTMB}\end{gathered} \tag{14}$$

$$\begin{gathered}\text{ID6} \sim \text{Age} + log(\text{SBS2/SBS13}) + \text{TP53} + log(\text{SBS2/SBS13}) \times \text{TP53} + (1|\text{Tumour Type}) \\ \text{Model family: Zero Inflated Negative Binomial Mixed Effects Model} \\ \text{R package: glmmTMB}\end{gathered} \tag{15}$$

CoxME Models

The function fitted by the CoxME package is summarised in [1] as:

$$\lambda(t) = \lambda_0(t)e^{X\beta + Zb}$$

$$b \sim G(0, \Sigma(\theta))$$

Here, t represents time, X represents the design matrix for fixed effects, and Z represents the design matrix for the random effects. $\lambda(t)$ represents the function that is being fit and $\lambda_0(t)$ represents a baseline hazard function. β represents a vector of coefficients for the fixed effects, and b represents a vector of coefficients for the random effects. G refers to the Gaussian distribution.

$$\text{Survival(Survival Time, Censoring Status)} \sim \text{SBS2/SBS13 Presence} + (1|\text{Tumour Type}) \tag{16a}$$

$$\text{Hazard}(t) = \text{Baseline Hazard Function}(t) \cdot e^{X \cdot \text{SBS} + Z \cdot \text{TT}} \tag{16b}$$

$$TT \sim G(0, \Sigma(\theta))$$

$$\text{Survival(Survival Time, Censoring Status)} \sim \text{SBS2/SBS13 Presence} \times \text{TP53 Mutation} + (1|\text{Tumour Type}) \tag{17a}$$

$$\text{Hazard}(t) = \text{Baseline Hazard Function}(t) \cdot e^{X \cdot (\text{SBS and TP53}) + Z \cdot \text{TT}} \tag{17b}$$

$$TT \sim G(0, \Sigma(\theta))$$

Supplementary Note 2: Fisher p-value combinations

For the comparison of the effect of APOBEC mutation presence or absence on genomic instability within tumour types, our hypothesis for all of the comparisons is the same, that the presence of SBS2 and SBS13 mutations results in increased genomic instaibility. For this reason, we used Fisher's combined probability test, to combine the p-values for each hypothesis test within individual tumour types [2].

References

[1] Therneau, T.M.: Package 'coxme' (2020). https://cran.hafro.is/web/packages/coxme/coxme.pdf Accessed 7 Apr 2022

[2] Fisher, R.A.: Statistical Methods for Research Workers. Oliver and Boyd, Edinburgh (1970)
